# Association between matrix metalloproteinase 9 C-1562T polymorphism and the risk of coronary artery disease: an update systematic review and meta-analysis

**DOI:** 10.18632/oncotarget.23293

**Published:** 2017-12-15

**Authors:** Ming-Ming Zhang, Xue-Wei Chang, Xue-Qin Hao, Hao Wang, Xiang Xie, Shou-Yan Zhang

**Affiliations:** ^1^ Department of Cardiology, Luoyang Central Hospital Affiliated to Zhengzhou University, Luoyang, PR China; ^2^ Department of Pharmacy, College of Animal Science and Technology, Henan University of Science and Technology, Luoyang, PR China; ^3^ Department of Cardiology, First Affiliated Hospital of Xinjiang Medical University, First Affiliated Hospital of Xinjiang Medical University, Urumqi, Xinjiang, PR China

**Keywords:** coronary artery disease, myocardial infarction, matrix metalloproteinase 9, polymorphism, meta-analysis

## Abstract

Polymorphism (rs3918242) in the MMP9 gene has been reported to be associated with coronary artery disease (CAD). This study aims to investigate a more accurate estimation of the relationship between CAD and rs3918242 polymorphism by a meta-analysis method. We systematically searched studies on the association of rs3918242 polymorphism and CAD in PubMed, Web of Science, the Cochrane Library, Wanfang Data and CNKI. We used Stata 12.0 and RevMan 5.3 software to perform the meta-analyses. A total of 37 case-control studies involving 13,035 CAD patients and 11,372 non-CAD controls were included. A statistically significant association between rs3918242 polymorphism and CAD was observed in allelic model (Odds ratio (OR) 1.34; 95% confidence interval (CI) 1.20–1.50; *p* < 0.00001), recessive model (OR 1.43; 95% CI 1.17–1.75; *p* = 0.0004), and in dominant model ( OR 1.36; 95% CI 1.20–1.53; *p* < 0.00001). Moreover, we also found that there is a statistically significant association between rs3918242 polymorphism and myocardial infarction (MI) in Asians with allelic model (OR 1.66; 95% CI 1.29–2.14; *p* < 0.0001), recessive model (OR 2.29; 95% CI 1.44–3.63; *p* = 0.004), and dominant (OR 1.74; 95% CI 1.29–2.35; *p* = 0.0003) model. A similar result in Caucasians with allelic model (OR 1.14; 95% CI 1.02–1.27; *p* = 0.02), and in dominant (OR 1.17; 95% CI 1.04–1.32; *p* = 0.01) model. Our meta-analysis suggested that the MMP9 T allele is a risk factor for CAD and MI.

## INTRODUCTION

Current studies have well documented that the interaction between various environmental factors and certain genetic polymorphisms may lead to CAD [1]. Many association studies between polymorphisms of matrix metalloproteases (MMPs) gene and CAD have been carried out [2, 3]. These studies showed that MMPs is associated with a higher risk of plaque rupture/atherosclerosis and adverse cardiovascular events in patients undergoing CAD [4].

Matrix metallopeptidases 9 (MMP9) has been focused on the value of degrade a wide range of extracellular matrix proteins in patients [5]. MMP 9 is regulated primarily at the transcription level and posttranslational by activation of the zymogen and by inhibition of the endogenous inhibitor TIMP-1[6]. Although various studies between MMP9 and CAD have been reported, the conclusions are not consistent. The MMP9 C-1562T (rs3918242) in the promoter region is of special interest, which was considered a close association with CAD by many studies. Up to now, lots of case-control studies and systematic reviews on the relation between rs3918242 and CAD were carried out. However, the conclusions were inconsistent. Based on these observations, to investigate a more accurate estimation of the relationship between CAD and rs3918242, we conducted an update meta-analysis.

## RESULTS

### Study characteristics

**Figure 1:**
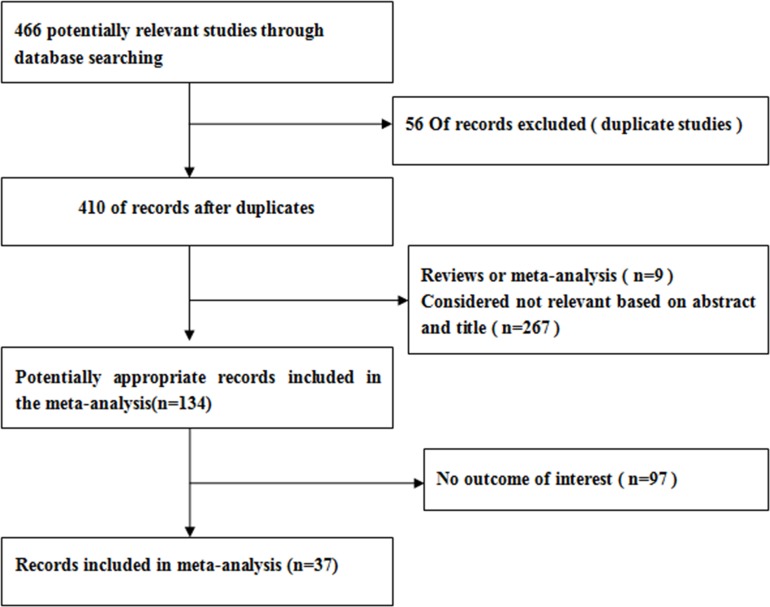
Flow chart of meta-analysis for exclusion /inclusion of individual studies

A total of 37 studies [7–41] including 13,035 cases and 11,372 controls were identified in this meta-analysis. The Figure 1 show that the study selection process. Supplementary Table 1 and Table 1 have summarized the main characteristics of included studies. In all studies, the genotype frequencies in controls were in consistent with HWE. The results of NOS showed that the methodological quality of be included studies were mostly good (6–9 stars).

**Table 1:** Characteristics of included studies

Reference	Year	Ethnicity	Case						Control					
			*N*	Genotype (*n*)			allele		*N*	Genotype (*n*)			allele	
				CC	CT	TT	C	T		CC	CT	TT	C	T
Wang et al.	2001	Caucasian	619	479	128	12	1086	152	169	128	41	0	297	41
Pollanen et al.	2001	Caucasian	109	78	21	10	177	41	167	124	30	13	278	56
Cho et al.	2002	Asian	63	48	15	0	111	15	67	63	4	0	130	4
Kim et al.	2002	Asian	131	99	32	0	230	32	117	85	32	0	202	32
Morgan et al.	2003	Caucasian	998	779	203	16	1761	235	265	223	42	0	488	42
Haberbosch et al.	2005	Caucasian	2098	1596	469	33	3661	535	633	485	132	16	1102	164
Chen et al.	2005	Asian	78	57	21	0	135	21	81	73	8	0	154	8
Tang et al.	2005	Asian	101	73	27	1	173	29	105	91	13	1	195	15
Meng et al.	2006	Asian	117	91	26	0	208	26	99	80	18	1	178	20
Nuzzo et al.	2006	Caucasian	115	73	39	3	185	45	123	86	36	1	208	38
Horne et al.	2007	Caucasian	1693	1219	440	34	2878	508	3455	2591	795	69	5977	933
Nanni et al.	2007	Caucasian	200	136	62	2	334	66	201	135	63	3	333	69
Chen et al.	2007	Asian	150	97	48	5	242	58	70	61	6	3	128	12
Chen et al.	2007	Asian	110	92	13	5	197	23	70	61	6	3	128	12
Wang et al.	2007	Asian	64	46	17	1	109	19	84	66	18	0	150	18
Koh et al.	2008	Asian	206	151	52	3	354	58	173	142	31	0	315	31
Zhang et al.	2008	Asian	92	67	22	3	156	28	95	83	12	0	178	12
Alp et al.	2009	Caucasian	146	99	42	5	240	52	122	90	29	3	209	35
Wu et al.	2009	Asian	791	628	155	8	1411	171	689	545	143	1	1233	145
Wu et al.	2009	Asian	370	289	77	4	655	85	689	545	143	1	1233	145
Fallah et al.	2010	Asian	145	77	57	11	211	79	157	62	76	19	200	114
Zhi et al.	2010	Asian	762	585	174	3	1344	180	555	442	110	3	994	116
Gao et al.	2010	Asian	96	49	38	9	136	56	78	59	18	1	136	20
Ma et al.	2010	Asian	347	251	83	13	585	109	403	346	53	4	745	61
Yong et al.	2010	Asian	128	97	30	1	224	32	106	92	14	0	198	14
Ghaderian et al.	2011	Asian	234	177	47	10	401	67	200	141	53	6	335	65
Wang et al.	2011	Asian	352	261	80	11	602	102	421	355	61	5	771	71
Opstad et al.	2012	Caucasian	996	756	225	15	1737	255	204	154	46	4	354	54
Wang et al.	2012	Asian	384	286	87	11	659	109	451	373	72	6	818	84
Spurthi et al.	2012	Asian	100	40	47	13	127	73	100	48	46	6	142	58
Han et al.	2012	Asian	91	65	25	1	155	27	101	75	25	1	175	27
Sewelam et al.	2013	Caucasian	40	32	7	1	71	9	40	40	0	0	80	0
Yang et al.	2013	Asian	240	186	47	7	419	61	200	161	35	4	357	43
Wu et al.	2013	Asian	258	193	56	9	442	74	153	131	22	0	284	22
Xu et al.	2013	Asian	382	268	109	5	645	119	466	361	103	2	825	107
Lu et al.	2014	Asian	168	102	62	4	266	70	208	156	50	2	362	54
Yuan et al.	2014	Asian	61	48	11	2	107	15	55	38	16	1	92	18

### Meta-analysis

Table 2 presents a principal results of this studies. For the rs3918242 polymorphism, heterogeneity was found in the allelic (I^2^ = 66%, *p* < 0.00001) and dominant (I^2^ = 65%, *p* < 0.00001) models, but not in the recessive model (I^2^ = 22%, *p* = 0.13). Therefore, We performed a random-effects and fixed-effects method to merge the ORs. The meta-analysis results showed that significant statistical association between rs3918242 polymorphism and the risk of CAD in allelic (OR 1.34; 95% CI 1.20–1.55; *p* < 0.00001), recessive (OR 1.43; 95% CI 1.17–1.75; *p* = 0.0004) and dominant (OR 1.36; 95% CI 1.20–1.53; *p* < 0.00001) models.

**Table 2:** Results From a meta-analysis of the association between CAD and matrix metalloproteinase 9 C-1562T polymorphism

Polymorphism and Subgroup			No. of Studies	No. of Cases	No. of Controls	Genotype					
						T/C		TT/CT+CC		CT+TT/CC	
						OR and 95% CI	*P* Value	OR and 95% CI	*P* Value	OR and 95% CI	*P* Value
**Ethnicity**	**All population**		37	13035	11372	1.34 (1.20, 1.50)	< 0.00001	1.43 (1.17, 1.75)	0.0004	1.36 (1.20, 1.53)	< 0.00001
	**Caucasian**		10	7014	5379	1.11 (0.99, 1.25)	0.07	1.06 (0.80, 1.40)	0.70	1.13 (1.01, 1.26)	0.03
	**Asian**		27	6021	5993	1.45 (1.25, 1.69)	< 0.00001	1.94 (1.45, 2.58)	< 0.00001	1.48 (1.25, 1.75)	< 0.00001
**Outcome**	**Caucasian**	CAD	6	4966	1560	1.08 (0.96, 1.23)	0.20	1.07 (0.71, 1.61)	0.75	1.09 (0.95, 1.26)	0.20
		MI	4	2048	3819	1.14 (1.02, 1.27)	0.02	1.05 (0.71, 1.55)	0.81	1.17 (1.04, 1.32)	0.01
	**Asian**	CAD	18	3799	3400	1.35 (1.12, 1.62)	0.002	1.73 (1.20, 2.51)	0.004	1.35 (1.11, 1.65)	0.003
		MI	9	2222	2593	1.66 (1.29, 2.14)	< 0.0001	2.29 (1.44, 3.63)	0.004	1.74 (1.29, 2.35)	0.0003

**Figure 2:**
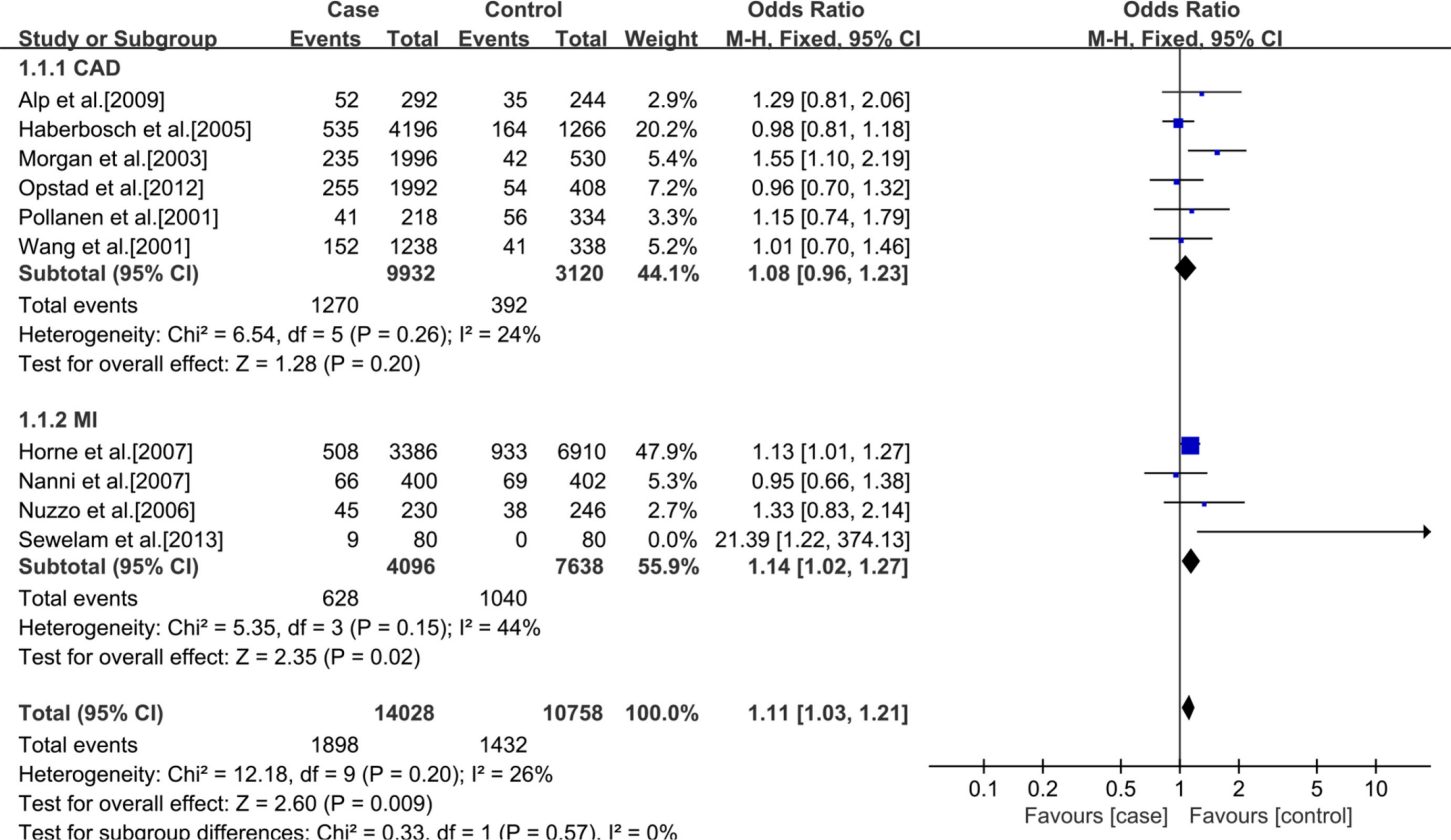
Forest plot of the meta-analysis of the association between MMP-9 C-1562T(rs3918242) and CAD or MI risks in an allele genetic model in Caucasians subgroup

**Figure 3:**
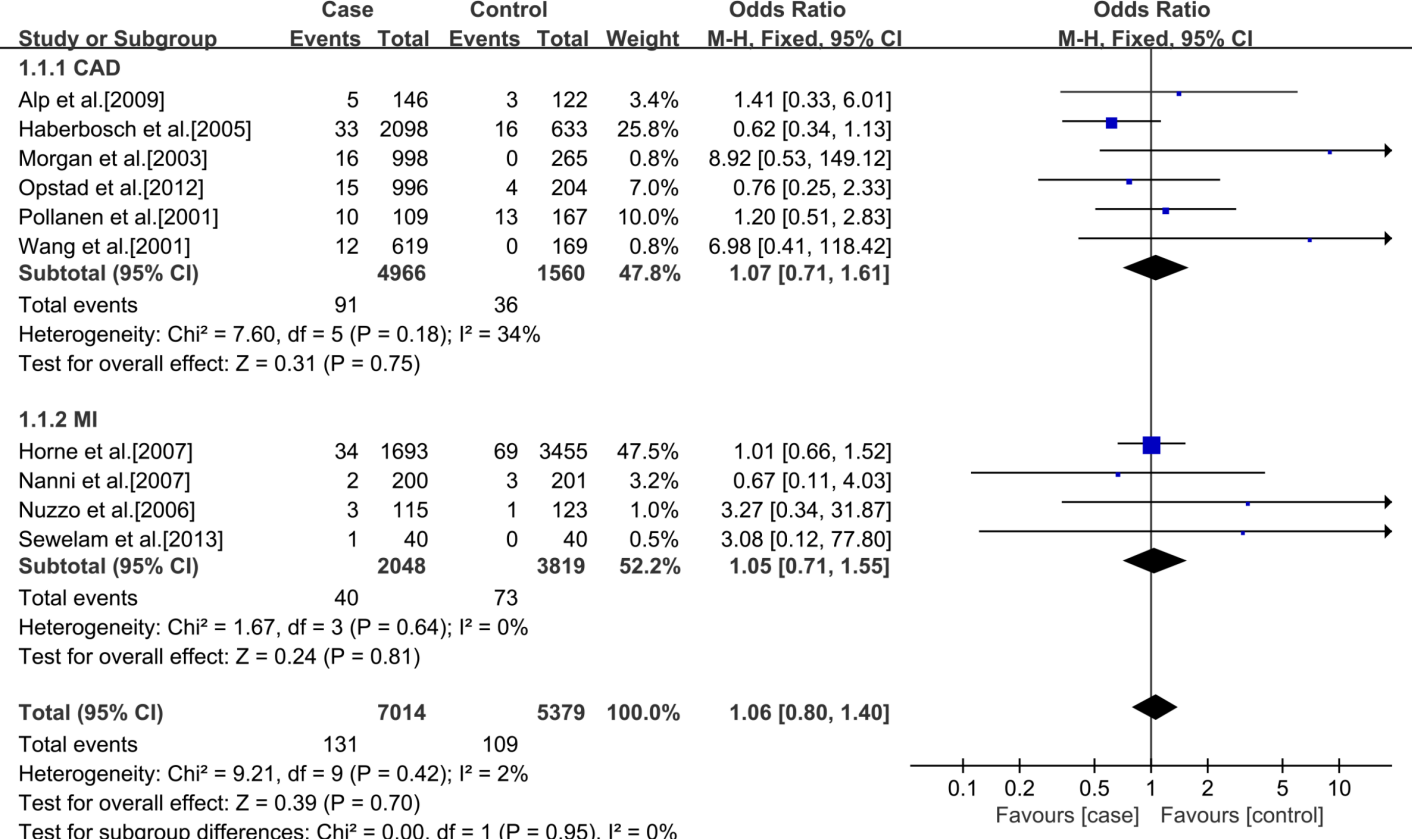
Forest plot of the meta-analysis of the association between MMP-9 C-1562T(rs3918242) and CAD or MI risks in a recessive genetic model in Caucasians subgroup

**Figure 4:**
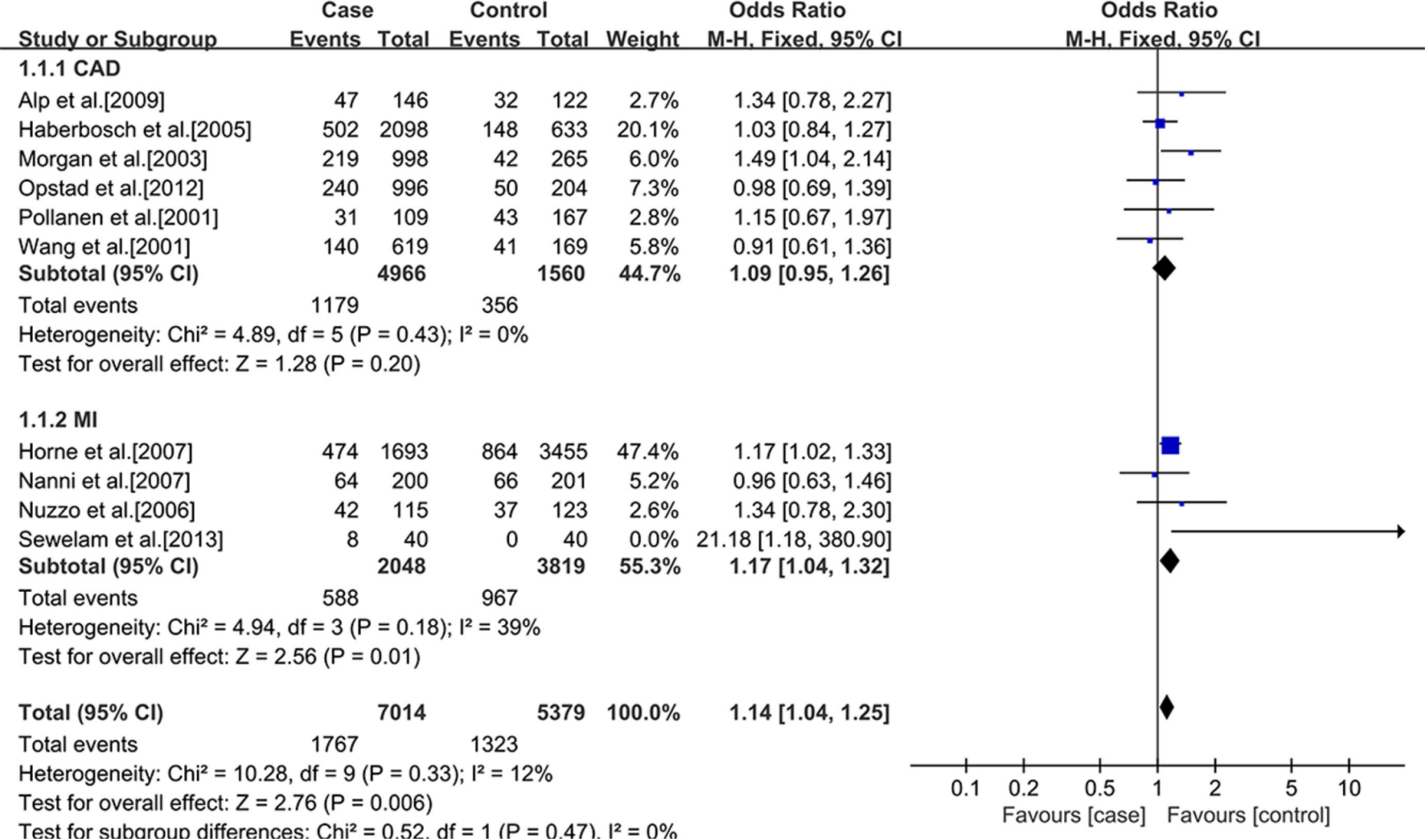
Forest plot of the meta-analysis of the association between MMP-9 C-1562T(rs3918242) and CAD or MI risks in a dominant genetic model in Caucasians subgroup

**Figure 5:**
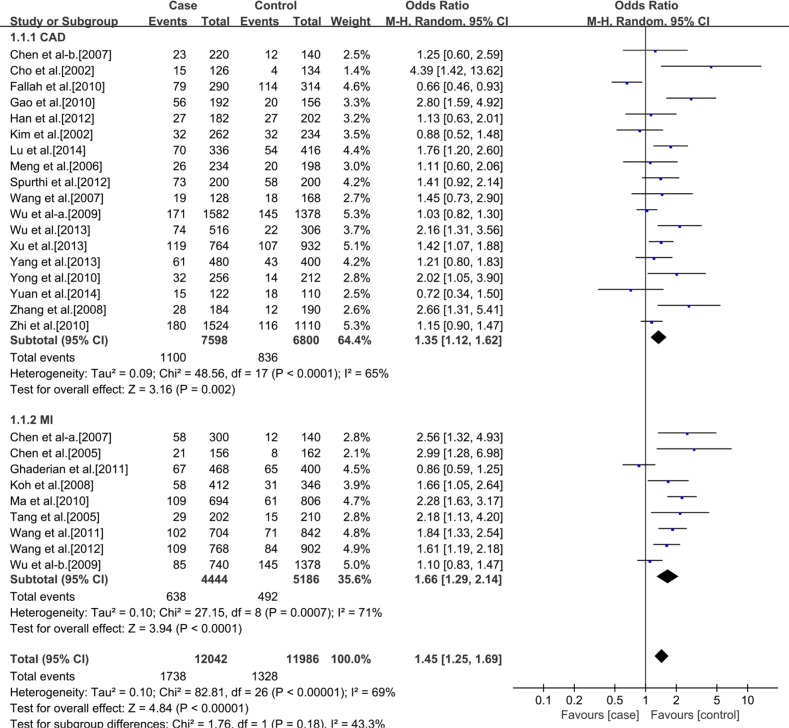
Forest plot of the meta-analysis of the association between MMP-9 C-1562T(rs3918242) and CAD or MI risks in an allele genetic model in Asians subgroup

**Figure 6:**
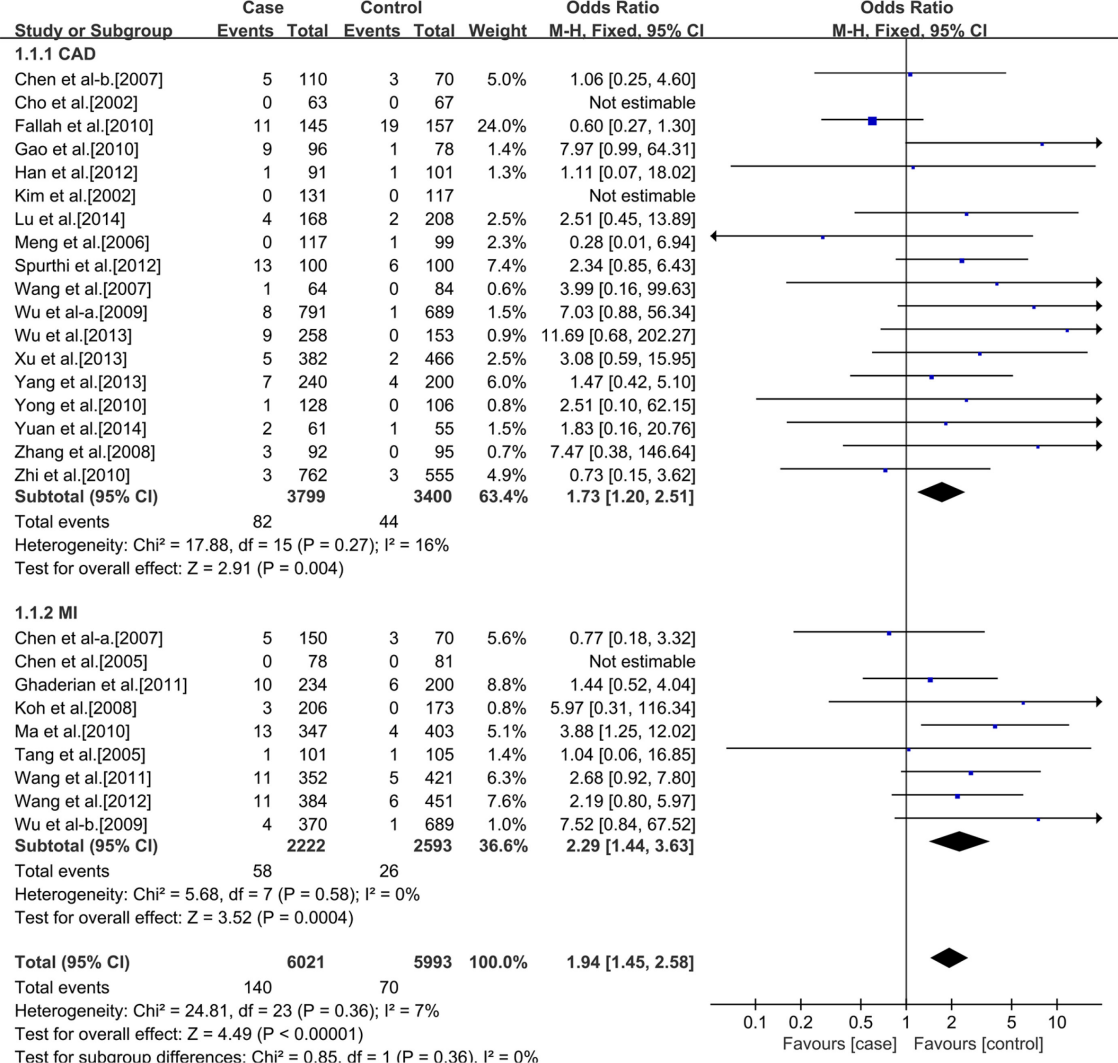
Forest plot of the meta-analysis of the association between MMP-9 C-1562T(rs3918242) and CAD or MI risks in a recessive genetic model in Asians subgroup

**Figure 7:**
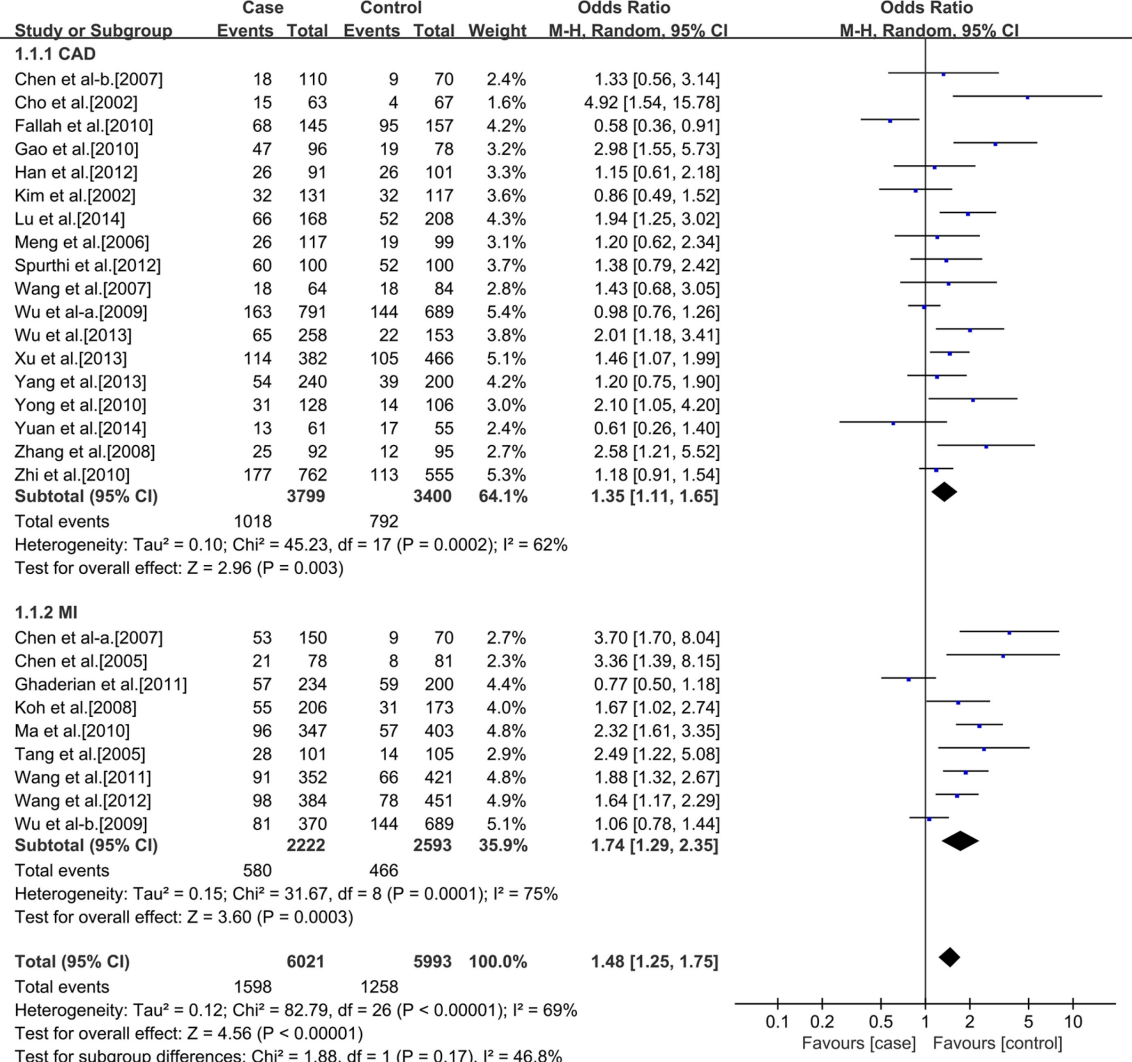
Forest plot of the meta-analysis of the association between MMP-9 C-1562T(rs3918242) and CAD or MI risks in a dominant genetic model in Asians subgroup

In addition, a subgroup analysis was conducted according to ethnics. In Caucasians, no significant statistical association between rs3918242 polymorphism and CAD either in allelic (OR 1.11; 95% CI 0.99–1.25; *p* = 0.07) or recessive (OR 1.06; 95% CI 0.80–1.40; *p* = 0.70) models. But significant statistical association was observed in dominant (OR1.13; 95% CI 1.01–1.26; *p* = 0.03) model. In Asians, significant statistical association was found between rs3918242 and CAD in allelic contrast (OR 1.45; 95% CI 1.25–1.69; *p* < 0.00001), recessive(OR 1.94; 95% CI 1.45–2.58; *p* < 0.00001) and dominant (OR 1.48; 95% CI 1.25–1.75; *p* < 0.00001) models (Figures 2–7).

### Sensitivity analyses

We excluded individual studies one at a time and recalculated the pooled *p* or OR for the remaining studies. The results proved that the ORs were not changed obviously, which suggested that this results are stable.

### Publication bias

**Figure 8:**
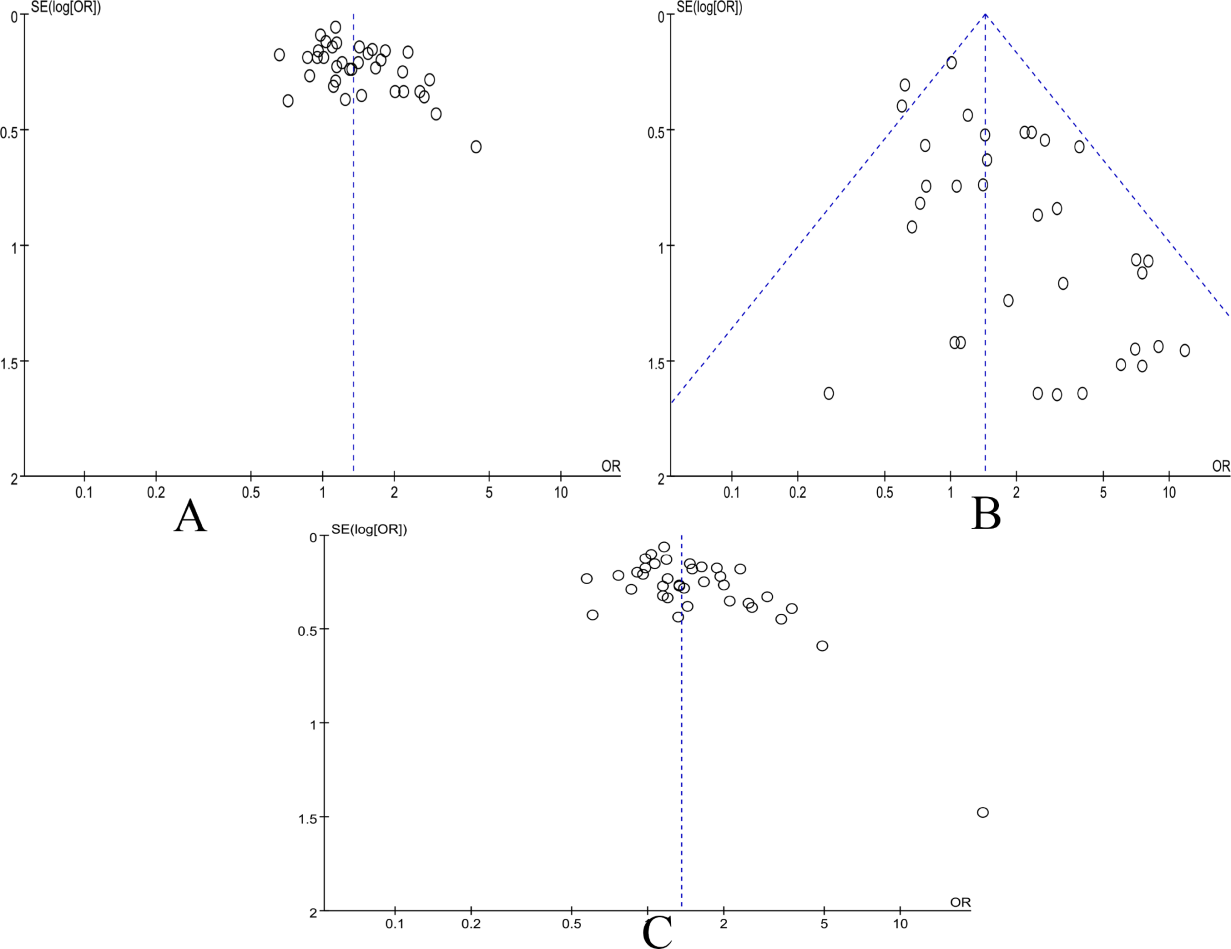
Funnel plot of the association between MMP-9 C-1562T(rs3918242) and CAD risk in all populations (**A**) The allele genetic model in all populations. (**B**) The recessive genetic model in all populations. (**C**) The dominant genetic model in all populations.

Egger’s test and Funnel plot were conducted to evaluate the publication bias of all contrast models. No obvious bias was found in our study. No obvious asymmetry was found in the funnel plot for the allelic, recessive and dominant genetic models (Figure 8). Further, Egger’s test be used to detect the whole publication bias. No statistically significant of publication bias was detected in allelic (*p* = 0.592), recessive (*p* = 0.103) and dominant (*p* = 0.683) models. The same was true in the subgroup analysis.

## DISCUSSION

Our meta-analysis showed that rs3918242 polymorphism was linked with an increased risk of CAD in Asians. More available evidence supports the fact that the rs3918242 as a risk factor association with MI both in Asian and Caucasian populations. MMP-9 might be particularly important in matrix degradation and the subsequent atherosclerotic plaque rupture because of its extensive substrate specificity and distal position in the proteolytic cascade reaction [42]. Loftus et al. reported that MMP-9 concentration and activity were significantly higher in unstable atherosclerotic plaque with intense inflammatory cell infiltration, hence contributing to the plaque rupture ultimately [43]. Opstad et al. suggested that patients with previous MI were associated with the higher MMP-9 gene expression [32]. The current study data from animal experiment, observation of circulating markers and expression investigations on atherosclerotic tissue, has indicated a role of MMP-9 in atherosclerosis. However, the association of rs3918242 polymorphism with CAD and MI risks remains inclusive, although several meta-analysis research (Wang et al. [42], Abilleira et al. [44], Juan et al. [45]) have been published. Wang [42] performed a meta-analysis which included sixteen case-control studies to evaluate the association between rs3918242 and CAD. Subgroup analysis was performed according to different races and outcome(CAD or MI). The final conclusion suggested that an obvious ethnic difference. MMP-9 C1562T polymorphism was associated with CAD or MI in East Asians. But not to west Asians or western populations. Abilleira [43] presented a study of available data from five studies and did not find association of MMP9 polymorphism with CAD. Also, they did not have further specific analysis and subgroup analysis. Juan [44] collected all publications on the association between rs3918242 polymorphism and MI which included 7 researchs. Their data showed that the rs3918242 is a risk factor for white populations, but not for Asian populations.

In order to obtain reliable conclusion, we implemented an update meta-analysis involving 37 studies to provide the relationship between rs3918242 and CAD or MI risks. The study revealed that rs3918242 possibly increased the risk of MI in both Asian and Caucasian populations. As compared with the former studies [43–45], our findings has a lot of novelty. Firstly, although lots of studies and systematic review have reported this association, the conclusions were inconsistent or inconclusive. Therefore, our research is urgent and meaningful. Secondly, our meta-analysis is superior to the others, due to the far larger number of participants (37 included studies with 13,035 cases and 11,372 controls) which were from all over the world. The substantially large sample size ensures the reliability of the results. Thirdly, subgroup analysis was further conducted according to different races.

There are several possible metabolic and molecular mechanisms to explain our conclusion. Zhang [46] have suggested that the C-1562T polymorphic locus is important for the regulatory element that the mutation appeared to be a binding site for a transcription repressor protein and T-allelic promoter had a higher promoter activity. Moreover, more evidences have indicated that the MMP-9 have associated with cell migration and proliferation [47]. More important, the overexpression and activity of MMP-9(rs3918242) was monitored in unstable atherosclerotic plaques [48]. These evidences were in line with our results.

Several limitations should to be pointed out. Risk of bias and unobservable heterogeneity may disturb the results. Included studies of language limit to English and Chinese, missing some studies by other languages. Some hardly to be avoided publication bias might exist. Such as some factors like age, gender, individual conditions, environment and experimental method are different, those might influence the interpretation of result in our meta-analysis.

In conclusion, the meta-analysis provided evidence that MMP9 rs3918242 polymorphism was significantly associated with CAD/ MI in Asian populations. The same of available evidence supports the fact that the rs3918242 is a risk factor for MI in Caucasian populations. Larger studies with the consideration of more influence factors and better study designs are still required to further evaluate the connection of MMP9 rs3918242 polymorphism with CAD/MI susceptibility.

## MATERIALS AND METHODS

### Literature search

All studies that researched the association between the rs3918242 polymorphism and CAD were identified by comprehensive computer-based searches of PubMed, Web of Science, the Cochrane Library, Wanfang Data and China National Knowledge Infrastructure (CNKI). The language was limited to English and Chinese articles before Feb.2016. The following keywords were used : #1 matrix metalloproteinase [MeSH]; #2 matrix metalloproteinase 9 [MeSH]; #3 polymorphism [MeSH]; #4 mutation [MeSH]; #5 variation [MeSH]; #6 genotype [MeSH]; #7 coronary artery disease [MeSH]; #8 coronary heart disease [MeSH]; #9 myocardial Infarction [MeSH]; #10 ischemic cardiovascular disease[MeSH]. The retrieval strategy is #1 or #2 and #3 or #4 or #5 or #6 and #7 or #8 or #9 or #10.

### Inclusion criteria

The diagnosis of CAD was fitted to the examination results of coronary arteriography, treadmill exercise test, clinical symptoms combined with electrocardiogram, as well as some other inspection project, for example echocardiography and myocardial perfusion imaging. The inclusion criteria for eligible studies were as follows: (1) independent case-control studies using either a hospital-based or a population-based design; (2) the studies of MMP9 C-1562T polymorphism and CAD risk; (3) the studies has an intact original data on genotype distribution and a comprehensive statistical index, sufficient data for estimating an odds ratio (OR) with 95% confidence interval (CI); (4) no repeat published data.

### Exclusion criteria

We excluded studies if (1) reviews, editorials, and articles with insufficient information; (2) the genotype distribution in the control group non-conformity the Hardy-Weinberg equilibrium.

### Data extraction

Two authors independently extracted the data. The extracted information included the first author’s name, publication year, study population, number of genotypes, genotyping methods, allele frequency of cases and controls, sample sizes in the cases and controls, sex and age of cases and controls. Disagreement was resolved by consensus. If these two authors could not reach a consensus, the result was reviewed by a third author.

### Quality assessment

To determine the methodological quality of the included studies, we used the Newcastle–Ottawa scale [49], which uses a “star” rating system to judge the quality of observational studies. The NOS ranges between zero (worst) up to nine stars (best). Two authors assessed the quality of included studies independently and solved disagreement through discussion.

### Statistical analysis

The association between rs3918242 polymorphism and CAD, which in our meta-analysis were compared by using the OR and its corresponding to 95% CI. Hardy–Weinberg equilibrium (HWE) was assessed by Chi-square test in control groups, and *P* < 0.05 was considered a significant departure from HWE. Heterogeneity between studies was assessed by I^2^ test, *p* < 0.10 and I^2^ > 50% indicated evidence of heterogeneity. If heterogeneity existed among the studies, the random effects model was used to estimate the pooled OR (the DerSimonian and Kacker method). Otherwise, the fixed effects model was adopted (the Mantel–Haenszel method) [50, 51]. The associations between the genetic variant and CAD risk of pooled ORs were performed for a recessive genetic model, dominant genetic model and allelic contrast. Z test was used to determine the pooled OR and significance was set at *p* < 0.05. Besides, subgroup analyses were stratified by ethnicity and outcome. The potential publication bias was checked by using funnel plots and Egger’s test [52]. The statistical analysis was performed by using Review Manager 5.30 (Cochrane Collaboration, The Nordic Cochrane Centre, Copenhagen) and Stata 12.0 software (StataCorp, College Station, TX, USA). A two-tailed *p* < 0.05 was considered significant.

## SUPPLEMENTARY MATERIALS AND TABLE





## References

[1] Yamada Y, Izawa H, Ichihara S, Takatsu F, Ishihara H, Hirayama H, Sone T, Tanaka M, Yokota M (2002). Prediction of the risk of myocardial infarction from polymorphisms in candidate genes. N Engl J Med.

[2] Kaplan RC, Smith NL, Zucker S, Heckbert SR, Rice K, Psaty BM (2008). Matrix metalloproteinase-3 (MMP3) and MMP9 genes and risk of myocardial infarction, ischemic stroke, and hemorrhagic stroke. Atherosclerosis.

[3] Medley TL, Cole TJ, Dart AM, Gatzka CD, Kingwell BA (2004). Matrix metalloproteinase-9 genotype influences large artery stiffness through effects on aortic gene and protein expression. Arterioscler Thromb Vasc Biol.

[4] Blankenberg S, Rupprecht HJ, Poirier O, Bickel C, Smieja M, Hafner G, Meyer J, Cambien F, Tiret L, AtheroGene Investigators (2003). Plasma concentrations and genetic variation of matrixmetalloproteinase 9 and prognosis of patients with cardiovasculardisease. Circulation.

[5] Newby AC (2005). Dual role of matrix metalloproteinases (matrixins) in intimal thickening and atherosclerotic plaque rupture. Physiol Rev.

[6] Visse R, Nagase H (2003). Matrix metalloproteinases and tissue inhibitors of metallopro-teinases: structure, function, and biochemistry. Circ Res.

[7] Wang J, Warzecha D, Wilcken D, Wang XL (2001). Polymorphism in the gelatinase B gene and the severity of coronary arterial stenosis. Clin Sci (Lond).

[8] Pöllänen PJ, Karhunen PJ, Mikkelsson J, Laippala P, Perola M, Penttilä A, Mattila KM, Koivula T, Lehtimäki T (2001). Coronary artery complicated lesion area is related to functional polymorphism of matrix metalloproteinase 9 gene: an autopsy study. Arterioscler Thromb Vasc Biol.

[9] Cho HJ, Chae IH, Park KW, Ju JR, Oh S, Lee MM, Park YB (2002). Functional polymorphism in the promoter region of the gelatinase B gene in relation to coronary artery disease and restenosis after percutaneous coronary intervention. J Hum Genet.

[10] Kim JS, Park HY, Kwon JH, Im EK, Choi DH, Jang YS, Cho SY (2002). The roles of stromelysin-l and the gelatinase B gene palymorphism in stable angina. Yonsei Med J.

[11] Morgan AR, Zhang B, Tapper W, Collins A, Ye S (2003). Haplotypic analysis of the MMP-9 gene in relation to coronary artery disease. J Mol Med (Berl).

[12] Haberbosch W, Gardemann A (2005). Gelatinase B C(−1562)T polymorphism in relation to ischaemic heart disease. Scand J Clin Lab Invest.

[13] Chen XF, Tang LJ, Zhu M, Jiang JJ, Shen WF, Du YX (2005). Matrix metalloproteinase-9 polymorphism (C1562T) and the susceptibility to myocardial infaction in Han population of China. Chin J Arterioscler.

[14] Tang LJ, Chen XF, Zhu M, Shen WF, Jiang JJ (2005). [Study of relations between matrix metalloproteinase-9 polymorphism (C-1562T) and acute coronary syndrome in Han population of China]. Zhonghua Yi Xue Yi Chuan Xue Za Zhi.

[15] Meng DM, Mao YM, Chen Q, Geng J, Qin Q, Zhao BR, Zhao FM, Cui RZ (2006). Relationship between polymorphisms of matrix metalloproteinase and coronary heart disease. Tianjin Med.

[16] Nuzzo D, Vasto S, Balistreri CR, Di-Carlo D, Listì F, Caimi G, Caruso M, Hoffmann E, Incalcaterra E, Lio D, Caruso C, Candore G Role of proinflammatory alleles in longevity and atherosclerosis: results of studies performed on -1562C/T MMP-9 in centenarians and myocardial infarction patients from Sicily. Ann N Y Acad Sci.

[17] Horne BD, Camp NJ, Carlquist JF, Muhlestein JB, Kolek MJ, Nicholas ZP, Anderson JL (2007). Multiple-polymorphism associations of 7 matrix metalloproteinase and tissue inhibitor metalloproteinase genes with myocardial infarction and angiographic coronary artery disease. Am Heart J.

[18] Nanni S, Melandri G, Hanemaaijer R, Cervi V, Tomasi L, Altimari A, Van Lent N, Tricoci P, Bacchi L, Branzi A (2007). Matrix metalloproteinases in premature coronary atherosclerosis: influence of inhibitors, inflammation, and genetic polymorphisms. Transl Res.

[19] Chen BY, Li XQ, He HJ, Chen XL, Li Q, Kuang XQ, He LP (2007). The relationship between matrix metalloproteinase-9 polymorphism (C1562T) and acute coronary syndrome. Chin J Arterioscler.

[20] Wang MF, Xiao CS, Gong SW, Wang RY, Liu XE (2007). Relationships study about polymorphism of matrix metalloproteinase-9 with coronary heart disease. J Clin Hematol.

[21] Koh YS, Chang K, Kim PJ, Seung KB, Baek SH, Shin WS, Lim SH, Kim JH, Choi KB (2008). A close relationship between functional polymorphism in the promoter region of matrix metalloproteinase-9 and acute myocardial infarction. Int J Cardiol.

[22] Zhang Y, Wang CX, Dong X (2008). Relationship between matrix metalloproteinase-2/9 polymorphism and susceptibility to premature coronary heart disease. J Xi'an Jiaot Univ.

[23] Alp E, Menevse S, Tulmac M, Kan D, Yalcin R, Erkan AF, Cengel A (2009). Lack of association between matrix metalloproteinase-9 and endothelial nitric oxide synthase gene polymorphisms and coronary artery disease in Turkish population. DNA Cell Biol.

[24] Wu N, Lu X, Hua Y, Song L, Ye J, Li J, Meng X, Gu D, Yang Y (2009). Haplotype analysis of the stromelysin-1(MMP3) and gelatinase B (MMP9) genes in relation to coronary heart disease. Ann Hum Genet.

[25] Fallah S, Seifi M, Ghasemi A, Firoozrai M, Samadikuchaksaraei A (2010). Matrix metalloproteinase-9 and paraoxonase1Q/R192 gene polymorphisms and the risk of coronary artery stenosis in Iranian subjects. J Clin Lab Anal.

[26] Zhi H, Wang H, Ren L, Shi Z, Peng H, Cui L, Ma G, Ye X, Feng Y, Shen C, Zhai X, Zhang C, Zen K (2010). Functional polymorphisms of matrix metallopeptidase-9 and risk of coronary artery disease in a Chinese population. Mol Biol Rep.

[27] Gao CX, Wang YL (2010). Research of the matrix metalloproteinase-9 gene polymorphism and risk of coronary heart disease. Mod Prev Med.

[28] Ma YT, Wang L, Xie X, Yang YN, Fu ZY (2010). Interactions between matrix metalloproteinase-9 polymorphism and hypertension in relation to myocardial infarction in a Chinese population. Chin J Hypertens.

[29] Yong FD (2010). The polymorphism in the MMP9 gene in relation to coronary heart disease. Journal of zhejiang university school of medicine.

[30] Ghaderian SM, Akbarzadeh Najar R, Tabatabaei Panah AS (2010). Genetic polymorphisms and plasma levels of matrix metalloproteinases and their relationships with developing acute myocardial infarction. Coron Artery Dis.

[31] Wang L, Yang YN, Fu ZY, Ma YT (2011). Association between matrix metalloproteinase-9 polymorphism (−1562C>T/R279Q) and acute coronary syndrome in Uygur nationality of Xinjiang Autonomous Region of China. Zhonghua Jizhen Yixue Zazhi.

[32] Opstad TB, Pettersen AA, Weiss TW, Akra S, Øvstebø R, Arnesen H, Seljeflot I (2012). Genetic variation, gene-expression and circulating levels of matrix metalloproteinase-9 in patients with stable coronary artery disease. Clin Chim Acta.

[33] Wang L, Ma YT, Xie X, Yang YN, Fu ZY, Li XM, Liu F, Huang Y, Ma X, Chen BD, Yuan S, Sun MH, Peng X, Wang BZ (2012). Interaction between MMP-9 gene polymorphisms and smoking in relation to myocardial infarction in a Uighur population. Clin Appl Thromb Hemost.

[34] Spurthi KM, Galimudi RK, Srilatha G, Sahu SK, Nallari P, Hanumanth SR (2012). Influence of gelatinase B polymorphic variants and its serum levels in atherosclerosis. Genet Test Mol Biomarkers.

[35] Han Y, Zhang Q, Su M, Zheng B (2012). Correlation between the matrix metalloproteinase-9 gene single nucleotide polymorphim and coronary artery stenosis degree. Zhongguo Laonianxue Zazhi.

[36] Sewelam NI, Radwan ER, Andraos AW, Ibrahim BE, Wilson MM (2013). Association between the polymorphisms of matrix metalloproteinases 9 and 3 genes and risk of myocardial infarction in Egyptian patients. Egypt J Med Hum Genet.

[37] Yang Y, Sun X, Zhu ZJ, Zhang YJ (2013). Study on the relationship between single nucleotide polymorphisms of MMP-9 gene and genetic predisposition of coronary disease. China Medical Herald.

[38] Wu HD, Bai X, Chen DM, Cao HY, Qin L (2013). Association of genetic polymorphisms in matrix metalloproteinase-9 and coronary artery disease in the Chinese Han population: a case-control study. Genet Test Mol Biomarkers.

[39] Xu X, Wang L, Xu C, Zhang P, Yong F, Liu H, Wang J, Shi Y (2013). Variations in matrix metalloproteinase-1, -3, and -9 genes and the risk of acute coronary syndrome and coronary artery disease in the Chinese Han population. Coron Artery Dis.

[40] Lu H, Hu DN, Peng LZ, Wang R (2014). Correlation between C1562T genetic polymorphism of MMP-9 and susceptivity of senile coronary heart disease. Chin J Evid Based Cardiovasc Med.

[41] Yuan C (2014). Relationship between Matrix Metalloproteinase-9 gene-1562C/T mutations with MMP-9 levels and coronary artery ectasia. Journal of Tianjin Medical University.

[42] Wang X, Shi LZ (2014). Association of matrix metalloproteinase-9 C1562Tpolymorphism and coronary artery disease: a meta-analysis. J Zhejiang Univ Sci B.

[43] Loftus IM, Naylor AR, Goodall S, Crowther M, Jones L, Bell PR, Thompson MM (2000). Increased matrix metalloproteinase-9 activity in unstable carotid plaques. A potential role in acute plaque disruption. Stroke.

[44] Abilleira S, Bevan S, Markus HS (2006). The role of genetic variants of matrix metalloproteinases in coronary and carotid atherosclerosis. J Med Genet.

[45] Juan Z, Wei-Guo Z, Heng-Liang S, Da-Guo W (2015). Association of Matrix Metalloproteinase 9 C-1562T Polymorphism with Genetic Susceptibility to Myocardial Infarction: A Meta-Analysis. Curr Ther Res Clin Exp.

[46] Zhang B, Ye S, Herrmann SM, Eriksson P, de Maat M, Evans A, Arveiler D, Luc G, Cambien F, Hamsten A, Watkins H, Henney AM (1999). Functional polymorphism in the regulatory region of gelatinase B gene in relation to severity of coronary atherosclerosis. Circulation.

[47] Pauly RR, Passaniti A, Bilato C, Monticone R, Cheng L, Papadopoulos N, Gluzband YA, Smith L, Weinstein C, Lakatta EG (1994). Migration of cultured vascular smooth muscle cells through a basement membrane barrier requires type IV collagenase activity and is inhibited by cellular differentiation. Circ Res.

[48] Davies MJ, Richardson PD, Woolf N, Katz DR, Mann J (1993). Risk of thrombosis in human atherosclerotic plaques: role of extracellular lipid, macrophage, and smooth muscle cell content. Br Heart J.

[49] Margulis AV, Pladevall M, Riera-Guardia N, Varas-Lorenzo C, Hazell L, Berkman ND, Viswanathan M, Perez-Gutthann S (2014). Quality assessment of observational studies in a drug-safety systematic review, comparison of two tools: the Newcastle–Ottawa scale and the RTI item bank. Clin Epidemiol.

[50] DerSimonian R, Kacker R (2007). Random-effects model for meta-analysis of clinical trials: an update. Contemp Clin Trials.

[51] Mantel N, Haenszel W (1959). Statistical aspects of the analysis of data from retrospective studies of disease. J Natl Cancer Inst.

[52] Egger M, Davey Smith G, Schneider M, Minder C (1997). Bias in meta-analysis detected by a simple, graphical test. BMJ.

