# Supplementary material for: Association between matrix metalloproteinase 9 C-1562T polymorphism and the risk of coronary artery disease: an update systematic review and meta-analysis
Source: Oncotarget. 2017 Dec 15;9(10):9468–79. doi: 10.18632/oncotarget.23293 (PMC5823656; doi:10.18632/oncotarget.23293)
Supplement: Supplementary file 1 [file oncotarget-09-9468-s001.pdf]

Association between matrix metalloproteinase 9 C-1562T polymorphism and the risk of coronary artery disease: an update systematic review and meta-analysis

SUPPLEMENTARY MATERIALS

Supplementary Table 1: Characteristics of included studies. See_Supplementary_Table 1
