# Supplementary material for: Association between matrix metalloproteinase 9 C-1562T polymorphism and the risk of coronary artery disease: an update systematic review and meta-analysis
Source: Oncotarget. 2017 Dec 15;9(10):9468–79. doi: 10.18632/oncotarget.23293 (PMC5823656; doi:10.18632/oncotarget.23293)
Supplement: Supplementary file 2 [file oncotarget-09-9468-s002.doc]

| **Supplementary Table 1: Characteristics of included studies** | | | | | | | | | | | | | | | |
| --- | --- | --- | --- | --- | --- | --- | --- | --- | --- | --- | --- | --- | --- | --- | --- |
| Reference | Year | Population | Eligible subjects  (n) | | Allele frequency (case) | |  | | | | | | | Genotyping  method | Selection  criteria |
|  |  |  |  |  | Case | Control | Male (n) | | Age (years) | | | NOS | HWE |  |  |
|  |  |  | Case | Control | T | T | Case | Control | Case | Control | *P* | star | *P* |  |  |
| Wang et al. | 2001 | Australia | 619 | 169 | 0.12 | 0.12 | NA | NA | 58±10 | 56±10 | ＞0.05 | 8 | 0.07 | PCR-RFLP | CAD |
| Pollanen et al. | 2001 | Finland | 109 | 167 | 0.19 | 0.17 | NA | NA | 61.3±7.9 | 59.3±8.5 | ＞0.05 | 7 | 0.11 | PCR-RFLP | CAD |
| Cho et al. | 2002 | Korea | 63 | 67 | 0.12 | 0.03 | 37 | 36 | ＜65 | ＜65 | ＞0.05 | 8 | 0.80 | PCR-RFLP | CAD |
| Kim et al. | 2002 | Korea | 131 | 117 | 0.12 | 0.14 | 89 | 59 | 58±9.01 | 55.3±10.2 | 0.04 | 7 | 0.08 | PCR-RFLP | CAD |
| Morgan et al. | 2003 | US | 998 | 265 | 0.12 | 0.08 | NA | NA | 63.2±10.4 | 61.3±9.32 | ＞0.05 | 9 | 0.16 | PCR-RFLP | CAD |
| Haberbosch et al. | 2005 | Germany | 2098 | 633 | 0.13 | 0.13 | NA | NA | 58.2±11.0 | 61.3±10.0 | 0.07 | 7 | 0.06 | PCR-RFLP | CAD |
| Chen et al. | 2005 | China | 78 | 81 | 0.13 | 0.05 | 57 | 58 | 56.4±7.7 | 54.3±8.1 | ＞0.05 | 8 | 0.64 | PCR-RFLP | MI |
| Tang et al. | 2005 | China | 101 | 105 | 0.14 | 0.07 | 73 | 86 | 64.1±10.7 | 62.3±11.2 | ＞0.05 | 7 | 0.49 | PCR-RFLP | MI |
| Meng et al. | 2006 | China | 117 | 99 | 0.11 | 0.10 | 40 | 32 | 64±12 | 63±12 | 0.443 | 7 | 0.99 | PCR-RFLP | CAD |
| Nuzzo et al. | 2006 | Italy | 115 | 123 | 0.20 | 0.15 | 109 | 100 | 47.8±6.2 | 47.0±5.5 | NA | 6 | 0.18 | PCR-RFLP | MI |
| Horne et al. | 2007 | US | 1693 | 3455 | 0.15 | 0.14 | 1253 | 2280 | 60.90±5.01 | 59.44±4.83 | ＞0.05 | 8 | 0.38 | TaqMan | MI |
| Nanni et al. | 2007 | Italy | 200 | 201 | 0.17 | 0.17 | 200 | 201 | 58±10 | 56±10 | 0.174 | 8 | 0.14 | PCR-RFLP | MI |
| Chen et al. | 2007 | China | 150 | 70 | 0.19 | 0.09 | 85 | 43 | 58.6±10.7 | 57.7±10.2 | ＞0.05 | 8 | 0.13 | PCR-RFLP | MI |
| Chen et al. | 2007 | China | 110 | 70 | 0.10 | 0.09 | 60 | 43 | 59.2±10.4 | 57.7±10.2 | ＞0.05 | 8 | 0.13 | PCR-RFLP | CAD |
| Wang et al. | 2007 | China | 64 | 84 | 0.15 | 0.11 | 43 | 52 | 63.08±13.0 | 62.55±9.2 | ＞0.05 | 7 | 0.27 | PCR-RFLP | CAD |
| Koh et al. | 2008 | Korea | 206 | 173 | 0.14 | 0.09 | 133 | 105 | 61.1±11.8 | 58.3±11.8 | 0.01 | 7 | 0.19 | PCR-RFLP | MI |
| Zhang et al. | 2008 | China | 92 | 95 | 0.15 | 0.06 | 62 | 59 | 54.5±6.4 | 52.3±6.9 | 0.452 | 7 | 0.51 | PCR-RFLP | CAD |
| Alp et al. | 2009 | Turkey | 146 | 122 | 0.18 | 0.14 | 88 | 59 | 59.30±9.1 | 57.30±9.7 | 0.085 | 8 | 0.71 | PCR-RFLP | CAD |
| Wu et al. | 2009 | China | 791 | 689 | 0.11 | 0.11 | 651 | 589 | 59.01±10.9 | 60.42±9.1 | ＞0.05 | 8 | 0.64 | PCR-RFLP | CAD |
| Wu et al. | 2009 | China | 370 | 689 | 0.11 | 0.11 | 311 | 589 | 57.70±11.4 | 60.42±9.1 | ＞0.05 | 8 | 0.64 | PCR-RFLP | MI |
| Fallah et al. | 2010 | Iran | 145 | 157 | 0.27 | 0.36 | 75 | 80 | 58.41±9.1 | 55.35±9.4 | NA | 7 | 0.55 | PCR-RFLP | CAD |
| Zhi et al. | 2010 | China | 762 | 555 | 0.12 | 0.10 | 543 | 372 | 67.46±9.6 | 69.90±11.4 | 0.4 | 9 | 0.16 | PCR-RFLP | CAD |
| Gao et al. | 2010 | China | 96 | 78 | 0.29 | 0.13 | 64 | 51 | 59.71±8.9 | 60.02±8.1 | ＞0.05 | 7 | 0.77 | PCR-RFLP | CAD |
| Ma et al. | 2010 | China | 347 | 403 | 0.16 | 0.08 | 192 | 229 | 55.56±10.9 | 54.11±10.3 | 0.11 | 8 | 0.22 | PCR-RFLP | MI |
| Yong et al. | 2010 | China | 128 | 106 | 0.13 | 0.07 | 74 | 65 | 64±10.5 | 61±11.2 | ＞0.05 | 7 | 0.46 | PCR-RFLP | CAD |
| Ghaderian et al. | 2011 | Iran | 234 | 200 | 0.14 | 0.16 | 188 | 111 | 59.40±3.6 | 60.8±5.9 | ＞0.05 | 8 | 0.70 | TaqMan | MI |
| Wang et al. | 2011 | China | 352 | 421 | 0.14 | 0.08 | 247 | 253 | 60.32±10.1 | 59.26±9.2 | 0.26 | 8 | 0.21 | PCR-RFLP | MI |
| Opstad et al. | 2012 | Norway | 996 | 204 | 0.13 | 0.13 | 778 | 140 | 62（36-81） | NA | NA | 6 | 0.79 | TaqMan | CAD |
| Wang et al. | 2012 | China | 384 | 451 | 0.14 | 0.09 | 255 | 291 | 55.6±10.9 | 54.1±10.3 | 0.116 | 7 | 0.24 | PCR-RFLP | MI |
| Spurthi et al. | 2012 | India | 100 | 100 | 0.36 | 0.29 | 74 | 54 | 56.73±12.3 | 54.55±14.4 | ＞0.05 | 7 | 0.24 | PCR-RFLP | CAD |
| Han et al. | 2012 | China | 91 | 101 | 0.15 | 0.13 | 60 | 67 | 61±11.12 | 59±10.23 | 0.231 | 7 | 0.48 | PCR-RFLP | CAD |
| Sewelam et al. | 2013 | Egyptian | 40 | 40 | 0.11 | 0 | 31 | 35 | 35-67（53.5） | 36-64（51.5） | ＞0.05 | 6 | 0.43 | PCR-RFLP | MI |
| Yang et al. | 2013 | China | 240 | 200 | 0.13 | 0.11 | 159 | 126 | 61.6±11.7 | 61.9±10.6 | ＞0.05 | 8 | 0.21 | PCR-RFLP | CAD |
| Wu et al. | 2013 | China | 258 | 153 | 0.14 | 0.07 | 146 | 83 | 63.97±12.32 | 63.61±11.8 | ＞0.05 | 8 | 0.33 | PCR-RFLP | CAD |
| Xu et al. | 2013 | China | 382 | 466 | 0.16 | 0.11 | 263 | 305 | 62±9 | 61±9 | 0.071 | 8 | 0.06 | PCR-RFLP | CAD |
| Lu et al. | 2014 | China | 168 | 208 | 0.21 | 0.13 | 96 | 120 | 73.64±8.78 | 71.67±8.69 | ＞0.05 | 8 | 0.35 | PCR-RFLP | CAD |
| Yuan et al. | 2014 | China | 61 | 55 | 0.12 | 0.16 | 44 | 37 | 58.4±4.8 | 59.1±6.8 | ＞0.05 | 7 | 0.64 | PCR-RFLP | CAD |
